# ﻿Phylogeny of the planthopper genus *Megamelus* (Hemiptera, Delphacidae), with the description of two new species from South America

**DOI:** 10.3897/zookeys.1224.135596

**Published:** 2025-01-21

**Authors:** Nicolas A. Salinas, Roxana Mariani, Ana M. Marino de Remes Lenicov, Marcela S. Rodriguero, Alejandro J. Sosa

**Affiliations:** 1 Fundación para el Estudio de Especies Invasivas (FuEDEI), Hurlingham, Buenos Aires, Argentina Fundación para el Estudio de Especies Invasivas (FuEDEI) Buenos Aires Argentina; 2 Consejo Nacional de Investigaciones Científicas y Técnicas (CONICET), CABA, Argentina Consejo Nacional de Investigaciones Científicas y Técnicas (CONICET) CABA Argentina; 3 Division Entomología, Facultad de Ciencias Naturales y Museo, Universidad Nacional de La Plata, Buenos Aires, Argentina Universidad Nacional de La Plata Buenos Aires Argentina; 4 Instituto de Ecología, Genética y Evolución de Buenos Aires (IEGEBA), Buenos Aires, Argentina Instituto de Ecología, Genética y Evolución de Buenos Aires (IEGEBA) Buenos Aires Argentina; 5 Departamento de Ecología, Genética y Evolución, FCEN-UBA, Buenos Aires, Argentina Departamento de Ecología, Genética y Evolución, FCEN-UBA Buenos Aires Argentina

**Keywords:** Distribution, host plant, phylogenetics, planthopper, species key, taxonomy

## Abstract

*Megamelus* is a genus of Delphacidae widely distributed and mostly associated with plants in freshwater environments. Despite various taxonomic revisions and thorough research, the delimitation of the genus, its diversity, and its evolutionary history need to be further explored. Moreover, features originally considered distinctive of the genus exhibit variation and should be reassessed. Here, the genus *Megamelus* in South America was examined, describing two new species, *Megamelusdelticus* Remes Lenicov & Mariani, **sp. nov.** and *Megamelusserpentinus* Mariani & Remes Lenicov, **sp. nov.**, and providing information on their host plants and geographical distribution. The distribution and host range knowledge of *Megamelusiphigeniae* and *Megamelustimehri* are also expanded, the male brachypter of *M.timehri* described for the first time, and a key to distinguish the species, based on male and female genitalia and their external morphology, is provided. Moreover, the first phylogenetic analysis of the genus is presented, based on the mitochondrial COI gene to clarify the interspecific relationships among its members. Our combined findings support the monophyly of the genus and refine diagnostic features, including the importance of the pygofer’s lobed appearance. This comprehensive revision highlights the need for further multidisciplinary approaches to fully understand the evolutionary history of *Megamelus* and its interactions with host plants and environments.

## ﻿Introduction

*Megamelus* Fieber, 1866 (Delphacinae: Delphacini) is a genus of planthoppers which includes 31 species widely distributed in the Holarctic (23 spp.) and Neotropical (7 spp.) regions and Australia (1 sp.), mostly associated with plants in freshwater environments (Mariani et al. 2013; Bartlett 2020). The genus was originally established to encompass two species from Europe, erected on the base of *Delphaxnotula* Germar, 1830, now *Megamelusnotulus* (Germar). A few years later, *M.scutellaris* Berg, 1883 was described from Argentina, being the first *Megamelus* species known in the Americas. Subsequently, several authors described a significant number of North American species and provided keys at a generic and specific level (Van Duzee 1897; Crawford 1914; Metcalf 1923; Muir and Giffard 1924; Beamer 1955). Nonetheless, most of the species were posteriorly transferred to other genera. The last revision of the genus north of Mexico was conducted by Beamer (1955), who made exhaustive taxonomic studies, described 11 new species, updated information for all the 20 recognized North American species, and proposed a new key to the genus. Moreover, he stated that the lobed appearance of the ninth segment of the male abdomen (pygofer) and other genito-anal structures were the main diagnostic features for this genus. The pygofer had already been recognized as a feature with diagnostic value, although complementary, by Muir and Giffard (1924). However, each of the structures that compose the male terminalia were described later by Muir (1926) for the South American species. Features such as color pattern and a narrow head with the vertex extending in front of the eyes, amongst others, originally defined by Fieber (1866) as distinctive of the genus, were later seen to show variations and need to be reassessed.

In South America, seven *Megamelus* species have been described to date (Berg 1883; Muir 1919, 1926; Sosa et al. 2007b; Mariani et al. 2013). One of them, *M.scutellaris*, was proposed as a biological control agent of water hyacinth, *Pontederiacrassipes* Mart. (Pontederiaceae) (Sosa et al. 2004, 2007a) and was then introduced in water bodies invaded by this aquatic weed in the United States, South Africa, and Argentina (Tipping et al. 2014; Coetzee et al. 2022). Research on *M.scutellaris*, which included field surveys in wetlands of South America with laboratory and field experiments, generated knowledge on its behavior and biology, with the discovery of two new *Megamelus* species, *M.bellicus* Remes Lenicov & Sosa, 2007 and *M.nigrifasciatus* Mariani & Remes Lenicov, 2013, and the redescription of *M.scutellaris*, *M.electrae* Muir, 1926, *M.iphigeniae* Muir, 1926, *M.timehri* Muir, 1919, and *M.maculipes* (Berg, 1879) (Sosa et al. 2004, 2007b; Mariani et al. 2013). These contributions provided descriptions of adults of both sexes and wing forms of all South American species, including biological observations and specific keys for males. For the first time, these keys included diagnostic features of the female genitalia, such as the relative length of the ovipositor, shape and position of valvifer VIII, and shape and denticulation of the first and second gonopophyses.

As with many other genera of Delphacidae, *Megamelus* still lacks standard revisionary studies including morphological, genetic, and ecological information, to better understand the biology and the phylogenetic relationships among species of the genus. In this study we describe two new *Megamelus* species and provide information on their biology and distribution. Additionally, we expand the distribution and host range of *M.iphigeniae* and *M.timehri*, and describe the male brachypter of *M.timehri* for the first time. We also perform the first phylogenetic analyses of the genus based on the mitochondrial COI gene to clarify the interspecific relationships among its members. To this end, we include most of the sequences of *Megamelus* species available to date in public databases. Finally, with this set of information, we expand the existing key to include the new species and wing forms of the South American species, expanding the range of morphological characters previously established as typical of the genus.

## ﻿Materials and methods

### ﻿Sample collection

Field surveys were conducted in Argentina and Paraguay between 2021 and 2023 in the search of *Megamelus* specimens. Our study encompassed sites across seven Argentine provinces (Buenos Aires, Entre Ríos, Corrientes, Misiones, Chaco, Formosa, and Santa Fe) and two departments from Paraguay (Cordillera and Presidente Hayes). Insects were sought after on plants previously cited in the literature as the hosts for the South American *Megamelus* species, mainly Pontederiaceae, Alismataceae, and Apiaceae, located in water bodies of public access such as rivers, streams, lagoons, marshes, and ditches. Surrounding plants were also surveyed.

Samples were collected directly from the host plant using insect aspirators. When possible, individuals were collected from plants located at ~ 5–10 meters apart, up to a total of four or five points per site to avoid sampling sibling insects. Samples were immediately placed in absolute ethanol and stored at -20 °C for morphological studies and DNA extraction. The species of *Megamelus* found were identified by the taxonomic criteria following Beamer (1955), Sosa et al. (2007b), and Mariani et al. (2013). *Megamelusnigrifasciatus* was the only species not found in the field during our surveys. Hence, this species was studied based on the holotype and other reference specimens deposited in the Museo de Ciencias Naturales de La Plata (**MLP**). Additionally, field collected samples of two North American species, *M.toddi* Beamer, 1955 and *M.hamatus* Beamer, 1955, were used for DNA extraction and phylogenetic analyses but were not included in the morphological studies.

Data generated in this study are accessible by the GenBank accession numbers PP986913–PP986946. Information for samples used in phylogenetic analyses, including collection dates and coordinates, host plant associations and accession numbers are shown in Suppl. material 1.

### ﻿Morphological studies

Males of the new species were described in detail, but only major differences were considered for females and the other winged forms. Both male and female genitalia were prepared for microscopic examination according to standard taxonomic techniques (Remes Lenicov and Virla 1993). The reported measurements come from five specimens of each sex and wing form and are given in millimeters. The male genitalia terminology mostly follows Asche (1985), but ‘genital styles’ is used instead of ‘parameres’, and ‘anal segment’ (segment X) and ‘anal style’ (segment XI) instead of ‘anal tube’. For descriptive purposes, the genital styles will be referred to distal ‘inner’ and ‘outer’ angles after Bartlett 2005 (sensu Metcalf 1949). ‘Genital complex’ is used to illustrate the set of aedeagus, connective, genital styles, and postgenital segments when these structures are separate from the pygofer. Nomenclature of carinae of the vertex follows Yang and Yang (1986). Photographs were taken using Leica EZ5 and Leica S9 D stereoscopic microscopes and a RRID 18 HD digital camera and a Canon EOS 90D reflex camera adapted to the microscope. Specimens were deposited in the collections of the **MLP**.

Abbreviations are used as follows: **L.**, total length; **B.L.**, body length; **b.w.**, body width; **M.b.w.**, maximum body width, **t.l.**, tegmina length; **v.l.**, vertex length; **v.w.**, vertex width at base; **f.l.**, frons length; **M.f.w.**, maximum frons width; **m.f.w.**, minimum frons width; **a.l.I**, first antennal segment length; **a.l.II**, second antennal segment length; **p.l.**, pronotum length; **m.l.**, mesonotum length; **mti.l.**, metatibia length; **mta.l.**, metatarsi length; **mta.Il.**, first hind tarsomere length; **s.l.**, metatibial spur length; and **t.n.**, number of teeth on metatibial spur; other measurements are relative.

Total length was measured from the anterior margin of vertex to the abdominal apex in brachypters, and up to the apex of the wings in macropters; body length was measured from the apex of vertex to the tip of abdomen in macropters; body width was measured in dorsal view at the external margin of tegulae. The length:width (L:W) ratio of the vertex was measured along the midline and near midlength, respectively. Averages are expressed as means ± standard error (SE).

Finally, a new key for the South American species, considering Beamer (1955), Sosa et al. (2007b), Mariani et al. (2013) and phylogenies derived from this study, is presented here to facilitate species identification. For the purposes of the proposed key only, we use the terms forewing and tegmen to refer to the first pair of wings of the macropters and brachypters, respectively.

### ﻿DNA extraction

Genomic DNA was extracted from whole bodies of adults of both sexes and wing forms of *M.scutellaris*, *M.bellicus*, *M.electrae*, *M.timehri*, *M.iphigeniae*, *M.maculipes*, *M.nigrifasciatus*, *M.serpentinus* sp. nov., *M.delticus* sp. nov., *M.toddi*, and *M.hamatus* using Qiagen DNeasy Blood & Tissue Kit according to the manufacturer’s instructions. After the lysis step, 2 µl of RNAse A were added and samples were incubated at 37 °C for 30 minutes. DNA concentration and quality was quantified using DS-11 Spectrophotometer/Fluorometer (Denovix) and visualized in 1% agarose gels stained with GelRed (Biotium).

### ﻿PCR amplification and Sanger sequencing

A fragment of 658 bp of the cytochrome c oxidase I (COI) gene was amplified using the primers LepF2_t1 and LepR1 (Park et al. 2011), extensively used for planthopper barcoding. Under the reaction conditions used for amplification, this primer pair also amplifies a bacterial sequence belonging to the genus *Rickettsia*, revealing the presence of this bacteria in many of the samples. Thus, for samples for which the primer pair LepF2_t1/LepR1 was not useful, the universal primer pair LCO/HCO (Folmer et al. 1994) was used instead.

PCR amplification was done in a 25 μL volume containing 16.95 μL of distilled water, 2.50 μL of 10 × reaction buffer, 0.75 μL of MgCl_2_ (50 mM), 2.50 μL dNTP mixture (4 mM), 0.6 μL of each primer (10 mM), 0.6 μL Taq DNA Polymerase and 1 μL of DNA. PCR thermocycling was performed under the following conditions: 2 min at 95 °C; 5 cycles of 40 sec at 94 °C, 40 sec at 45 °C, 1 min at 72 °C; 35 cycles of 40 sec at 94 °C, 40 sec at 51 °C, 1 min at 72 °C; 5 min at 72 °C; held at 4 °C. PCR products were checked in agarose gels and purified by adding 0.5 μL (10 u) Exonuclease I (Exo I) and 1 μL (1 u) Shrimp Alkaline Phosphatase (SAP). Samples were incubated at 37 °C for 15 min and the reaction was stopped by heating the mixture at 85 °C for 15 min. Sanger sequencing of the samples was done at Macrogen services (Korea) with the same primers used for PCR amplification. Posterior quality check and primer trimming were performed on CodonCode Aligner v. 10.0.2 (CodonCode Corporation). Alignment of sequences was performed using the MUSCLE algorithm as implemented in MEGA11 (Tamura et al. 2021), with default settings.

### ﻿Phylogenetic analyses

Phylogenetic relationships among *Megamelus* species were inferred by maximum likelihood (ML) analysis performed on W-IQ-TREE (Trifinopoulos et al. 2016) with 10,000 ultrafast Bootstrap alignments. HKY+I+R was chosen as the best-fit nucleotide substitution model for the dataset using ModelTest-NG (Darriba et al. 2020). Sequences from other genera were included as outgroups based on the positioning of *Megamelus* within the tribe Delphacini according to Urban et al. (2010). COI sequences from North American and European *Megamelus* and from other related genera were retrieved from the GenBank and BOLD databases: *M.davisi* Van Duzee, 1897 (BOLD: CNCHG1265-12, BBHMA1871-12), *M.notulus* (BOLD: ZMBN1987-21), *M.metzaria* Crawford, 1914 (KR034487.1, KR042817.1), *M.inflatus* Metcalf, 1923 (KR032845.1), *M.flavus* Crawford, 1914 (KR041514.1), *M.lunatus* Beamer, 1955 (KR034315), *M.distinctus* Metcalf, 1923 (KR033356.1, KR035000.1), *Stobaeratricarinata* (Say, 1825) (KR034279.1), *Bostaeraballi* Penner, 1952 (CNCHG1224-12), *Delphaxcrassicornis* (Panzer, 1796) (HEMFI929-15), *Peregrinusmaidis* (Ashmead, 1890) (ASIHE1417-12), *Euidesbasilinea* (Germar, 1821) (MZ631889.1), *Conomelusanceps* (Germar, 1821) (MZ631894.1, HEMFI926-15), and *Pissonotusparaguayensis* Bartlett, 2000 (OR523788.1). The PCR reactions performed with DNA obtained from dry specimens of *M.nigrifasciatus* failed to amplify; hence, this species was not included in the analysis.

## ﻿Results

### ﻿Taxonomy

#### ﻿ Megamelus delticus

Taxon classificationAnimaliaHemipteraDelphacidae

Remes Lenicov & Mariani sp. nov.

74A9ACBE-0114-5958-AA39-8C67EFC6E210

https://zoobank.org/2FCBA2D6-C16D-4006-BA70-CF3ADCB898E7

Figs 1 , 2 , 3

##### Type material.

***Holotype*** male (brachypter): Argentina • Buenos Aires, Otamendi, 08-VI-2022, on *Eryngium* sp., Salinas-Sosa cols. ***Paratypes*** • same data as holotype, 7 male brachypters, 6 female brachypters (MLP).

##### Other material.

Argentina • 6 male brachypters, 6 female brachypters, Buenos Aires, Otamendi, 08-VI-2022, on *Eryngium* sp., Salinas-Sosa cols. (MLP) • 1 male brachypters, 3 female brachypters, Buenos Aires, Dique Lujan, 19-VII-2023, on *Eryngium* sp., Salinas-Sosa cols. (MLP).

##### Type locality.

Argentina, Buenos Aires: Otamendi, Campana, 34.1818S, 58.8706W, forested river margin, on *Eryngium* sp., 8 August 2022.

##### Diagnosis.

Brachypter. The salient features of this new species include the following: dorsally overall dull dark brown color, with pale mottles on apex vertex, front disc, and a pale yellow stripe on frontoclypeal suture extending towards the base of gena. Body broadly depressed and distinctively wide at abdomen. Vertex broad, subquadrate, apical margin broadly rounded, with submedian carina forking dorsally near anterior margin of eyes, carinal branches diverging widely to meet anteriorly just below fastigium which is angled when viewed laterally. Eyes reduced, reddish, slightly emarginate below, barely visible in ventral view. Frons subcircular, short, about as long as wide, with lateral carinae bowed outward, converging both ventrally and dorsally; metatibial spur short and narrow, bearing eight or nine black-tipped sharp teeth on trailing margin. Male terminalia: pygofer short, with small sized outer lobes, inner lobes subtriangular in outline, with broad concavity between them; aedeagus short, bearing dorso-apical horseshoe-like process; anal segments short and wide, unarmed.

##### Description.

**Figure 1.**
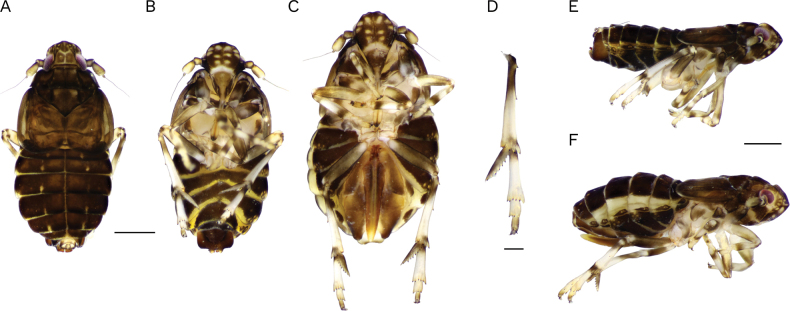
*Megamelusdelticus* sp. nov. Habitus. Brachypterous male and female. **A** male dorsal view **B** male ventral view **C** female ventral view **D** apex of hind leg (post tibial and tarsi) **E** male lateral view **F** lateral view. Scale bars: 0.5 mm (**A–C, E, F**); 0.2 mm (**D**).

**Figure 2.**
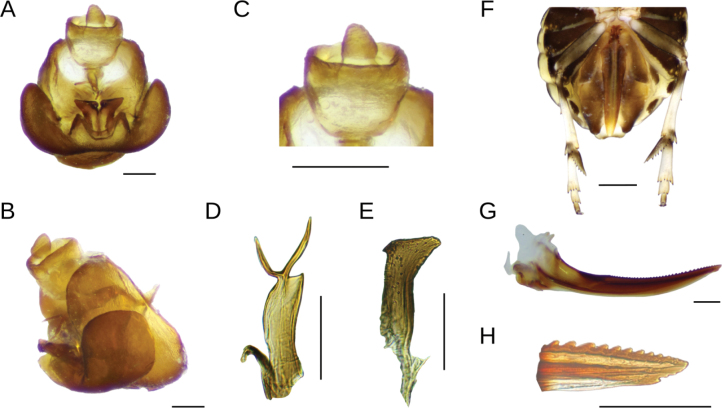
*Megamelusdelticus* sp. nov. Terminalia. Male: pygofer **A** posterior ventral view **B** lateral view **C** anal segment **D** aedeagus, dorsal view **E** right genital style. Female **F** abdomen ventral view **G** gonapophysis IX, lateral view **H** apex of gonapophysis IX. Scale bars: 0.1 mm (**A–E, G, H**); 0.5 mm (**F**).

**Brachypterous male** (Figs 1A, B, D, E, 2A–E). ***Color*** (Fig. 1) dull dark brown, with some pale marks. Reddish eyes. Vertex pale along posterior margin, median and Y-shaped carinae, with small yellowish spots on apex between submedian and lateral carina and also between lateral carina and eyes. Frons with continuous row of ~ 10 symmetrical pale dots paralleling median and lateral carinae, and transversal whitish stripe on frontoclypeal suture extending towards the base of gena. Clypeus castaneous on disc, rostrum yellowish. Antennal segments castaneous, slightly pale on anterior surface. Pronotum with pale transversal row of small spots on anterior margin between lateral carinae and several smaller spots on central disc near posterior margin; mesonotum disc with pale longitudinal spots between lateral carinae and some small spots on posterior margin. Tegmen uniformly pale brown. Legs yellowish, darker on base and apex of pro and mesocoxae, annular dark brown stripes near the base and apex of pro- and mesotibiae, apex of pro- and mesofemur, dorsal metafemur, and two annular stripes on metatibiae, one near base and the other on base of apical spines, on dorsal surface of spurs, and base of first tarsomere and apex of third. Abdomen dark brown in dorsal view, with longitudinal bilateral narrow pale stripes on tergites III–VII and ventrally on posterior margins of sternites IV–VII, and laterally around wax pores; anal segment paler, darker on apical margin as well as on anal style.

***Structure*.** Body strongly dorsoventrally flattened, suboval in outline. Head narrower than pronotum. Vertex in dorsal view almost as long as wide, rather quadrate, broadly rounded on anterior margin; basal compartment occupying approximately more than basal half of vertex. Median carina present, forked near anterior margin of eyes, arms of fork diverging strongly (angle 170°) to meet submedian frontal carinae. Submedian frontal carinae arising from the lightly foliate lateral carinae at level of middle of eyes, meeting anteriorly just at the fastigium (Fig. 1A). In lateral view, head projected downwards in front of the eye, fastigium angled (Fig. 1E). Frons subcircular, about as long as wide, and as long as clypeus; carinae of frons distinct, evanescent toward apex, lateral carinae bowed outward, converging both ventrally and dorsally; frons widest at antenna level. Frontoclypeal suture ventrally curved. Clypeus sub-triangular with carinae evident, the laterals continuing with genal carina. Rostrum reaching metacoxae, slightly shorter than frons plus clypeus, subapical segment longer than the apical one (1.3:1). Compound eyes, very reduced, lower margin only slightly incised, barely visible in ventral view. Antennae short, first segment as long as wide, second segment 2 × the first, 2 × longer than wide (Fig. 1A, B). Pronotum with conspicuous carinae, the laterals divergent, reaching hind margin and ending slightly convex. Mesonotal disc almost as long as pronotum (1.2:1), carinae conspicuous, lateral ones slightly divergent apically reaching hind margin (Fig. 1A). Tegmen coriaceous, subquadrate, posterior margin subtruncated to slightly rounded, reaching 4^th^ segment; veins distinct (Fig. 1A). Metatibial spur leaf-like, short, narrow apically and concave ventrally, bearing eight or nine black-tipped sharp teeth on trailing margin, almost as long as first segment of metatarsi at notch; first hind tarsomere longer than second plus third (1.7:1) (Fig. 1D). Abdomen broadest across segment V, decreasing in width towards apex (Fig. 1A).

***Terminalia*.** Pygofer trapezoidal, with laterodorsal margin slightly truncate, not projected caudad; ventrally ~ 2 × longer than dorsally; dorsally with shallow concave anal margination (Fig. 2A, B); outer lobes small-sized and rounded, slightly enfolding lateral area of pygofer, in ventral view occupying half the length of pygofer, inner lobes subtriangular in outline, broadly concave between them and with narrow notch between inner and outer lobes, partially closing ventral foramen (Fig. 2A, B); diaphragm fairly long, regularly narrowed toward middle line and caudally produced in short conical process. Aedeagus short, length-wide ratio: 2.2:1, regularly tubular and caudally downwardly directed, bearing an apical process projected on dorsal surface to left of phallotreme; this process is forked near base in two long and divergent semi-circularly curved spines (horse-shoe outline), in lateral view extending beyond genital styles (in repose); phallotreme large, near apex on dorsal surface to the right (Fig. 2D). Suspensorium sclerotized, strap-like, very short, half of aedeagus length (Fig. 2D). Genital styles (parameres) straight and flattened, widest and divergent in apical 1/3, apices truncate and enlarged, with sharp conical process on inner angle, widely expanded and rounded on outer angle, apex reaching dorsal margin of diaphragm at rest (Fig. 2E). Anal segment broad, collar-like, closely embraced by pygofer, without processes (Fig. 2C); anal style short (Fig. 2C).

***Measurements*** (*n* = 3). L., 2.6; b.w., 0.75; M.b.w. at abdominal segment V, 1; t.l., 0.4; v.l., 0.4; v.w., 0.5; f.l., 0.4; M.f.w., 0.4; m.f.w., 0.25; a.l.I, 0.15; a.l.II, 0.2; p.l., 0.3; m.l., 0.4; mti.l., 0.8; mta.l., 0.7; mta.Il., 0.4; s.l., 0.4; t.n., 9.

**Macropter** unknown.

**Brachypterous female** (Figs 1C, F, 2F–H). ***Color*.** Body coloration pattern and structure similar to male; ovipositor brown with valvifer pale on inner margin; gonapophysis rather pale apically.

***Terminalia*.** Ovipositor short, reaching anal segment at base (Fig. 2F). Valvifer VIII regularly broad, slightly excavated on inner margin near base, with inconspicuous basal projection; separate in repose in ventral view. Gonapophysis VIII wide at base, ventrally projected between valvifers. Gonapophysis IX slightly curved, bearing numerous strong rounded teeth (~ 35) on dorsal margin on > 1/2 of its length, a few teeth smaller distally; with three or four ventral teeth (Fig. 2G, H).

***Measurements*** (*n* = 3). L., 3; b.w., 0.83; M.b.w. at abdominal segment V, 1.265; t.l., 0.4; v.l., 0.35; v.w.,0.5; f.l., 0.4; M.f.w., 0.4; m.f.w., 0.25; a.l.I, 0.15; a.l.II, 0.2; p.l., 0.3; m.l., 0.4; mti.l., 0.85; mta.l, 0.75; mta.Il., 0.5; s.l., 0.4; t.n., 9–10.

**Macropter** unknown.

##### Etymology.

The specific name comes from the Greek letter delta (Δ), which was used to refer to the triangle of fertile land that the Nile forms at its mouth (Nile Delta) and by extension, to other river deltas. In this case, the name refers to the geographical distribution of the species, which is restricted to the region of the Paraná River Delta.

##### Distribution.

**Figure 3.**
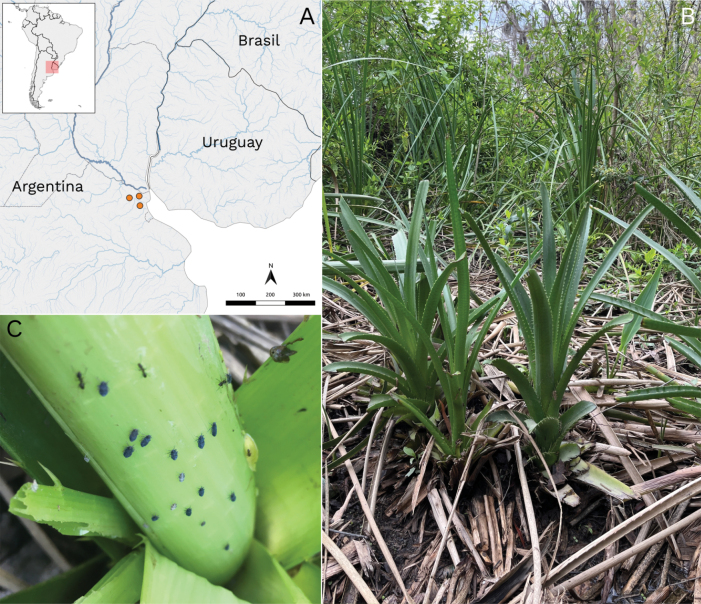
Geographical distribution and habitat of *Megamelusdelticus* sp. nov. **A** distribution map in Argentina, orange dots represent sites where *M.delticus* was found (Suppl. material 2) **B** habitat and host plants (*Eryngium* sp.) **C** adults and nymphs on *Eryngium* sp.

Argentina: Buenos Aires Province (Fig. 3A, Suppl. material 2).

##### Host plant.

*Eryngium* sp. (Apiaceae).

##### Ecology.

This planthopper was recorded in Otamendi and Dique Lujan, Paraná River Delta, in Buenos Aires Province (Argentina). It was only collected on *Eryngium* sp., a plant growing on the higher areas of river banks, where it is protected from periodical floods (Fig. 3B). Large numbers of nymphs and adults were found in the center of the plant mat (Fig. 3C), where leaves tend to accumulate water. The specimens collected had an abundant serous secretion covering their bodies, probably to repel the accumulated water. It is worth noting that *M.delticus* and *M.nigrifasciatus* were both sought after during our campaigns in search of *Megamelus* sp. and were only found in the same restricted geographical region, which suggests two possible cases of endemism.

##### Remarks.

This new species is easily distinguished from all the other *Megamelus* species by the broadly depressed body with a broad, sub quadrate vertex, large basal compartment, fastigium angled when viewed laterally, short and subcircular frons, small compound eyes, and the male pygofer slightly enfolded by the small sized outer lobes and the aedeagus ending in a horseshoe-like bifurcation. The dull dark brown coloration with pale dots and a transversal white stripe on the face, are also distinctive. Among the South American species, *M.delticus* and *M.nigrifasciatus* share the flat frons with convex lateral margins, the short and narrow spur with a few sharp teeth, brachypterism as the only wing form, and the short gonapophysis in females. Moreover, these species share their host plant (*Eryngium* sp.), which suggests that these morphological traits are likely adaptations to their ecological niche.

#### ﻿ Megamelus serpentinus

Taxon classificationAnimaliaHemipteraDelphacidae

Mariani & Remes Lenicov sp. nov.

AC267F4A-FD44-5382-8525-F4647ADC8549

https://zoobank.org/DDA5B411-4039-4D88-815C-B9B9E8239FCE

Figs 4 , 5 , 6

##### Type material.

***Holotype*** male (macropter): Argentina • Corrientes, Esquina, -29.99197098266, -59.52115137130, V-2022, on *Pontederiaazurea*, Salinas-Sosa col. (MLP). ***Paratypes*** • same data as holotype, 3 macropterous males (1 with genitalia dissected), 5 macropterous females, 2 brachypterous females, 2 brachypterous males (MLP).

##### Other material.

Argentina • 1 male macropter, Santa Fe, Reconquista, 26-XI-1939, Biraben-Bezzi (MLP); • 1 female macropter, Chaco, Resistencia, 20-III-1939, Denier, col. (MLP); • 1 female brachypter, Misiones, Concepción de la Sierra, 27-XI-2022, on *Pontederiaazurea*, Salinas col. (MLP); • 1 male brachypter, Buenos Aires, Arroyo Botija, 10-VI-2023, on *Pontederiaazurea*, Salinas col. (MLP); • 2 female macropters, Corrientes, Ramada Paso, 15-V-2022, on *Pontederiaazurea*, Salinas col. (MLP); • 2 female macropters, Corrientes, Bañado Virocay, 27-XI-2022, on *Pontederiaazurea*, Salinas col. (MLP); Paraguay: • 2 female macropters, Cordillera, Arroyos y Esteros, 7-IV-2022, on *Pontederiaazurea*, Salinas col. (MLP).

##### Type locality.

Argentina, Corrientes: Esquina, -29.9920S, -59.5212W, on *Pontederiaazurea* floating near the bank of a stream, 12 May 2022.

##### Diagnosis.

Macropter and brachypter. The salient features of the new species include the following: body mostly dark brown with distinctive yellowish to white marks bordering most of the sclerites of the body with legs paler and lightly marked with dark pigment. Macropters with forewings amber with pale brown veins, with strong dark marks on clavus, along Cu vein, over cross veins, and on last apical cells; brachypter with tegmen amber, brownish transverse marks in middle and claval apex, male pygofer dark brown, with dorsal surface, anal angles, and anal segment pale brown. Vertex narrow, with submedian carina forking dorsally quite far from fastigium; carinal branches closely forming a slender triangle; fastigium rounded in lateral view. Frons long, median carina prominent at or just below fastigium then fine; lateral carinae at base foliated, all carinae fine before the well-defined frontoclypeal suture. Spur large and wide, with 17 or 18 sharp, black-tipped teeth on trailing margin. Male terminalia: pygofer with relatively large lobes, the inner sharpened at apex with rounded external margin and internal sinuous; aedeagus short and tubular, with a short, slender dorso-caudally process curved at apex. Anal segment with two long, sinuous, slender caudally directed processes projecting laterally from the base.

##### Description.

**Figure 4.**
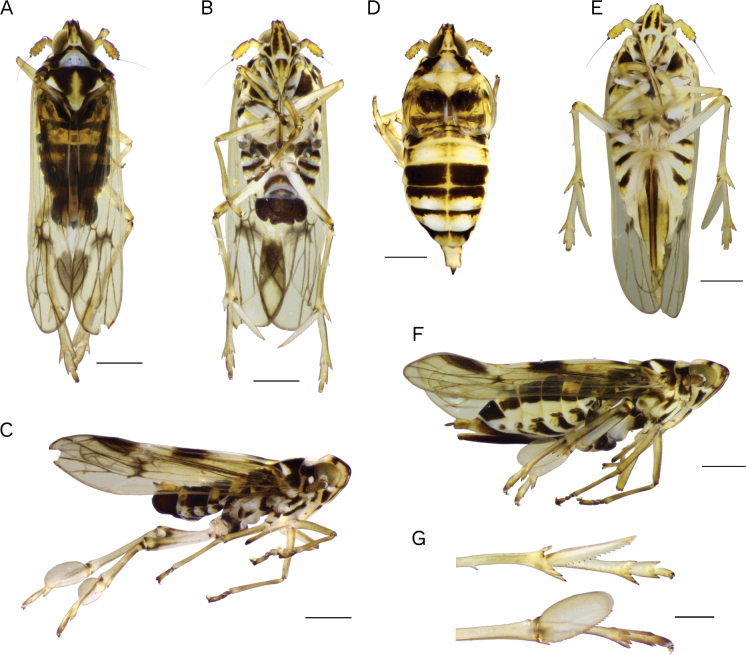
*Megamelusserpentinus* sp. nov. Habitus. Macropterous male and female. Male macropter **A** dorsal **B** ventral and **C** lateral view **D** female brachypter dorsal view **E** female macropter ventral and **F** lateral view **G** apex of hind leg (post tibial spur and tarsi) ventral and lateral view. Scale bars: 0.5 mm (**A–F**); 0.2 mm (**G**).

**Figure 5.**
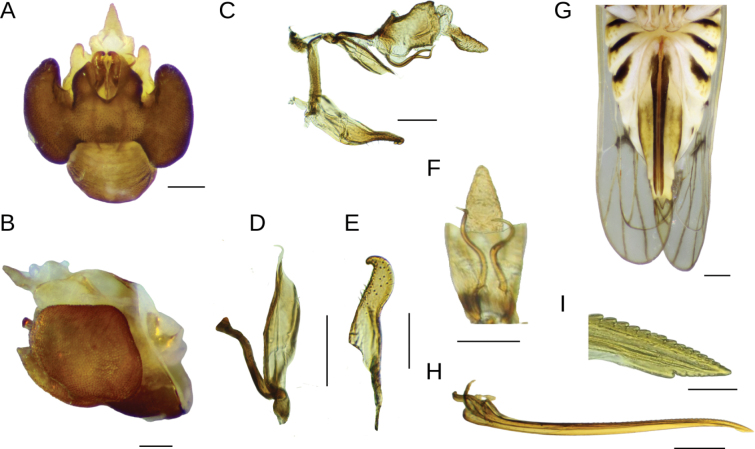
*Megamelusserpentinus* sp. nov. Terminalia. Male: pygofer **A** posterior ventral view **B** lateral view **C** genital complex, lateral view **D** aedeagus and suspensorium, lateral view **E** right genital style **F** anal segment, ventral view. Female **G** abdomen ventral view **H** gonapophysis IX, lateral view **I** apex of gonapophysis IX. Scale bars: 0.1 mm (**A–F, I**); 0.2 mm (**G, H**).

**Macropterous male** (Figs 4A–C, G, 5A–F). ***Color*** (Fig. 4A–C) dark brown, with distinctive marginal castaneous and yellowish white marks on head, thorax, and abdomen. Head yellowish on posterior compartments of vertex and fovea, only infuscated in concavities, and on both sides of lateral and median carinae of face below fastigium; distinctive whitish marks below the eyes and across frontoclypeal area. Thorax, whitish colored on the pronotal disc between lateral carinae, one spot behind eyes on paranotal disc, a subtriangular shaped spot on each side of postero-lateral margin of mesonotum, metanotum, and scutellum; yellowish, on lateral edges of pronotum, tegula, and a suboval longitudinal median spot on mesonotum disc. Antennal segments pale castaneous with basal segment and proximal 1/2 of second segment darkish on dorsal surface. Legs yellow with tarsi dorsally darker, with longitudinal darker spots near base and apex of pro- and mesocoxae, on dorsal surface of femora, apical region of metafemur, and base and apex of metatibiae. Forewing amber, veins pale brown, fuscous along apical veins M1+2 and M3+4 with infuscate areas on central nodal line, last apical cell, along Cu vein, on postclaval angle, and apex of clavus. Abdomen in dorsal view with contrasting white yellowish coloration on drumming segments and two lateral spots on tergites V and VIII, ventrally with most of the segment margined with yellow. Pygofer dark brown on outer lobes and ventral surface; pale castaneous on dorsal surface and inner lobes. Anal segment yellowish and anal style dark brown (Fig. 4A, B).

***Structure*.** Head narrower than pronotum. Vertex rectangular, longer than wide at base (2.1: 1) projecting beyond eyes > 1/3 of its length, with rounded frontal inflection; basal compartment slightly concave, occupying approximately more than basal third, stem of Y-shaped carina fine, delimiting shallow depressed areas on both sides; other carinae of head pronounced; submedian carinae forking at dorsal surface of vertex, quite far from fastigium, carinal branches forming a slender triangle, fovea, or areola little > 2 × the length; median carina strongly ridged and prominent at base at or just below fastigium then smooth; lateral carinae foliated at base, removed from eye along the length. (Fig. 4A). Frons nearly 3 × longer than wide (3:1.1), strongly narrowed between anterior margins of eyes, maximum width near basal 1/3, lateral margins slightly convex at apex; lateral and submedian carinae fine just before frontoclypeal suture, which is arched dorsally. Clypeus subtriangular, longer than wide, median carina weaker at base. Eyes globose, deeply emarginate below to receive the antennae. Rostrum reaching metacoxae, longer than frons, subapical and apical segment subequal. Antennae with the first segment longer than wide, the second segment < 2 × the first, length > 2 × its width (Fig. 4A, B). Pronotum with conspicuous carinae, laterals divergent, slightly convex toward hind margin, reaching it. Mesonotum almost as long as vertex plus pronotum, fine median carina becoming obsolete at apex, lateral carinae inconspicuous, slightly divergent posteriorly, not reaching hind margin (Fig. 4A). Forewings rather long and slender, rounded at apex, length 3 × their width at subapical region, surpassing distal end of abdomen > ~ 1/3 of their length. Metatibial spur, leaf-like, long, and broad, with median rib becoming obsolete at apex which is truncated without teeth, slightly longer than metatarsomere I, with 17 or 18 regular, large, black-tipped teeth on trailing margin; first hind tarsomere longer than second plus third (1.5:1) (Fig. 4G). Abdomen regularly wide, compressed dorsoventrally (Fig. 4A).

***Terminalia*** (Fig. 5A–F). Pygofer in dorsal view, with deeply concave anal emargination, anal angles distinctly projected caudad. In ventral view, expanded, with large, round, kidney-shaped outer lobes enfolding almost the entire lateral surface (Fig. 5A, B); inner lobes, large, wide in basal 2/3, external margin rounded, inner margin sinuous ending apically pointed with deep and broad concavity between their bases; strong emargination between inner and outer lobes (Fig. 5A, B); diaphragm broad with dorsal margin deeply concave and medially caudad projected into lip-shaped process bearing bunch of rather short, stiff hairs. Aedeagus short, tubular, strongly narrow at base, widening up to the basal 1/3 then uniformly tubular and obliquely truncated at apex, with a single short, slender, apically curved process extending shortly beyond oval and apical phallotreme (Fig. 5C, D). Genital styles (parameres) long, narrow, convergent apically, expanded and gradually tapering basally, broadly rounded along apical 1/2 on outer margin, apex hook-like, very curved inward; in ventral view visible between the internal lobes, in almost its entire length (Fig. 5E). Suspensorium strap-like, connected to the aedeagal base, as long as half length of aedeagus (Fig. 5C). Connective slightly compressed, almost straight (Fig. 5C). Anal segment tubular, longer than twice the width, with caudal margin deeply emarginate ventrally; with two long, sinuous, slender, caudally directed processes arising ventrolaterally just below anterior angle which is membranous; anal style slender, twice longer than broad (Fig. 5F).

***Measurements*** (*n* = 6). L., 3.8; B.L., 2.3; b.w., 1; t.l., 3.2; v.l., 0.4; v.w., 0.2; f.l., 0.6; M.f.w., 0.3; m.f.w., 0.2; a.l.I, 0.15; a.l.II, 0.3; p.l., 0.2; m.l., 0.5; mti.l., 1.13; mta.l., 1; mta.Il., 0.7; s.l., 0.8; t.n., 17–18.

**Brachypterous male. *Color*** similar to macropterous form, with mesonotum paler and uniformly colored; tegmen amber with veins concolorous, with fuscous transverse marks continuous or fragmented, from base of clavus at near axillary region, and one spot at claval apex. Abdomen with similar patterns except tergites V and VI which are more uniformly dark brown contrasting with yellowish segments VII and VIII.

***Structure*.** Mesonotum shorter, > 1/2 of vertex plus pronotum length. Tegmen slightly longer than wide, rounded on external lateral margins; posterior margin truncate, covering tergite II.

***Measurements*** (*n* = 6). L., 2.3; b.w., 0.8; t.l., 1; v.l., 0.4; v.w.,0.18; f.l., 0.6; M.f.w., 0.25; m.f.w., 0.15; a.l.I, 0.15; a.l.II, 0.3; p.l., 0.2; m.l., 0.25; mti.l., 0.9; mta.l., 0.9; mta.Il., 0.6; s.l., 0.65; t.n., 17–18.

**Macropterous female** (Figs 4E, F, 5G–I). ***Color***: Head and thorax resemble male. Abdomen, in dorsal view, with contrasting yellowish marks on sides and posterior margins of sclerites, except V and VIII, which are uniformly brown. Abdominal sternites with yellowish margins; pygofer and anal segment yellowish, anal style castaneous; ovipositor dark brown (Fig. 4E, F).

***Structure*.** Resembling male but abdomen is sharply tapered towards genital segments. Forewings surpassing distal end of abdomen ~ 1/6 of their length. Anal segment subrectangular; anal style slender.

***Terminalia*** (Fig. 5G–I). Pygofer long, tubular-shaped, tapering toward the apical 1/2; in dorsal view exposed shortly beyond tergite VIII. Ovipositor long, strong, slightly sinuous in apical 1/2, as long as length of pygofer plus anal segment. Valvifer VIII regularly wide, inner margin rounded at base, separated in repose (Fig. 5G). Gonapophysis IX, long and slender, overall shape slightly sinuous, apical fifth concave ventrally, with numerous blunt small teeth on most of dorsal surface (Fig. 5H, I).

***Measurements*** (*n* = 10). L., 4.3; B.L., 3.2; b.w: 0.9; t.l., 3.5; v.l., 0.45; v.w., 0.2; f.l., 0.7; M.f.w., 0.3; m.f.w., 0.2; a.l.I, 0.2; a.l.II, 0.3; p.l., 0.2; m.l., 0.6; mti.l., 1.1; mta.l., 1; mta.Il., 0.7; s.l., 0.6; t.n., 21–23.

**Brachypterous female** (Fig. 4D). ***Color*** pattern similar to that of the macropterous female, but tegmina resemble those of brachypterous male.

***Measurements*** (*n* = 5). B.L., 2.9; b.w: 0.8; t.l., 1; v.l., 0.45; v.w.,0.2; f.l., 0.7; M.f.w., 0.3; m.f.w., 0.2; a.l.I, 0.2; a.l.II, 0.3; p.l., 0.2; m.l., 0.3; mti.l., 1; mta.l, 1; mta.Il., 0.6; s.l., 0.7; t.n., 21–23.

##### Etymology.

The specific name comes from the Latin, *serpentinum* (serpentine), referring to the undulated shape of the long and slender male anal processes.

##### Distribution.

**Figure 6.**
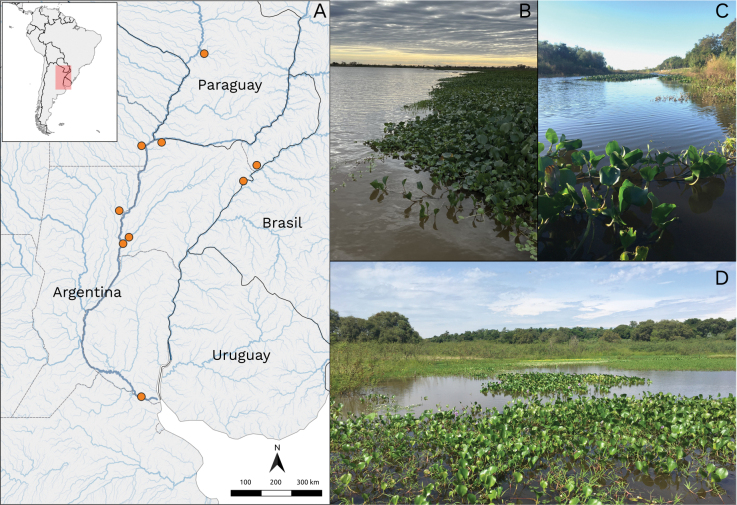
Geographical distribution and habitat of *Megamelusserpentinus* sp. nov. **A** distribution map in Argentina and Paraguay, orange dots represent sites where *M.serpentinus* was found (Suppl. material 2) **B–D** habitats and host plant (*P.azurea*).

Argentina: Misiones, Chaco, Corrientes, Santa Fe, and Buenos Aires provinces. Paraguay: Cordillera department (Fig. 6A, Suppl. material 2).

##### Host plant.

*Pontederiaazurea* Sw.

##### Ecology.

In the field, *M.serpentinus* was recorded on *P.azurea* in wetlands of the La Plata Basin (Fig. 6B–D). Both adults and nymphs were observed feeding on the host plant.

##### Remarks.

This species is distinguished from the other South American *Megamelus* species principally by their characteristic coloration and the morphology of the male genitalia. Salient features include whitish yellow marks on head, thorax and abdomen, forewings with strongly dark marks on clavus, along Cu vein, over cross veins and last apical cells; the slender vertex with submedian carinae forking at dorsal surface, quite far from fastigium; male with inner lobes of the pygofer long and sharpened at apex with a strong emargination among inner and outer lobes; the aedeagus short and tubular with a short thin apical process slightly curved apically, and the long anal segment with a long, slender and sinuous, posteriorly directed anal processes, emerging from its anterior ventral margin.

**Figure 7.**
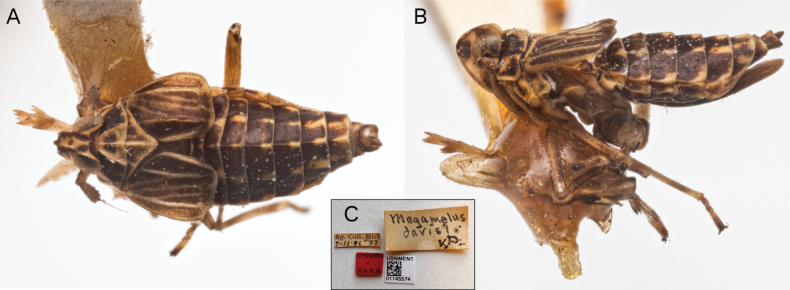
*Megamelusdavisi* Van Duzee female brachypter. Cotype (NHNM): Habitus **A** dorsal view **B** lateral view **C** labels.

Among its congeners, *M.serpentinus* shares morphological features with *M.davisi such as* the sinuous shape and placement of the anal processes in the male, the long ovipositor in the female and the large foliaceous spur, truncated at apex. The examination of the type specimens, macropter and brachypter, in the NHNM collection, by AMRL (Fig. 7) showed that *M.davisi* has a darker coloration, almost piceous black with the carinae and fine edges on sides and posterior margin of pro and mesothorax pale, the forewings whitish with only claval area darkish, and the tegmina darker with pale veins (in the brachypter). *Megamelusdavisi* also differs in the submedian carinae of vertex which is forked just on fastigium, and the flat shape of the aedeagus with a narrow twisted apex (Beamer 1955:32, plate 1, fig. 1). *Megamelusserpentinus* also shares with *M.timehri* and *M.davisi* the arrangement of the anal segment processes, caudally projected from the anterior ventral margin of the segment.

#### ﻿ Megamelus timehri

Taxon classificationAnimaliaHemipteraDelphacidae

Muir, 1919

15A12A37-53CA-5BD8-9876-235915B05678

Fig. 8

##### Material examined.

Argentina • 2 male brachypters, 2 female brachypters, Corrientes, Esteros del Iberá, 30-XI-2021, on *N.indica*, Salinas-Sosa cols; Paraguay • 2 male macropter, 5 male brachypter, 2 females brachypter, Cordillera, Arroyos y Esteros, 7-IV-2022, on *N.indica*, Salinas-Sosa cols. (MLP).

##### Description.

**Figure 8.**
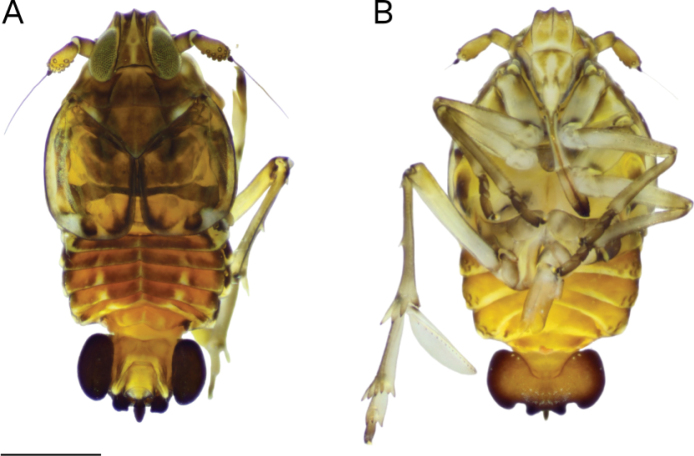
*Megamelustimehri Muir*. Habitus. Brachypterous male **A** dorsal view **B** ventral view. Scale bar: 0.5 mm (**A, B**).

**Brachypterous male. *Color*** (Fig. 8A, B) pattern dark brown lighter on dorsal pygofer and ventrally, similar to macropterous male; tegmina, amber-colored infuscation along claval margin and longitudinal veins, with a white spot on external apical corner.

***Structure*.** Tegmen slightly longer than wide, rounded on external lateral and posterior margins, covering tergite II (Fig. 8A).

***Measurements*** (*n* = 3). L., 2.12; b.w., 0.72; M.b.w. at abdominal segment V, 0.90; t.l., 0.74; v.l., 0.32; v.w., 0.18; f.l., 0.44; M.f.w., 0.25; m.f.w., 0.15; a.l.I, 0.13; a.l.II, 0.17; p.l., 0.19; m.l., 0.28; mti.l., 0.73; mta.l., 0.75; mta.Il., 0.46; s.l., 0.48; t.n., 20.

##### Distribution.

Argentina: Corrientes province (Sosa et al. 2007b). Guyana: Demerara River (Muir 1919). New record: Paraguay, Arroyos y Esteros Department.

##### Ecology.

*Megamelustimehri* was recorded during spring 2003 on *Limnobiumlaevigatum* (Humb. & Bonpl. ex Willd.) Heine (Hydrocharitaceae) in northeastern Argentina (La Plata Basin, subregión Iberá System) (Sosa et al. 2007b). *Nymphoidesindica* (L.) Kuntze is recorded as a new host plant. During our surveys (2021–2023) *M.timehri* was found abundantly and exclusively on this plant species across different sites. The previous record could be the result of *M.timehri* casually hopping and resting on *L.laevigatum*.

#### ﻿ Megamelus iphigeniae

Taxon classificationAnimaliaHemipteraDelphacidae

Muir, 1926

59E2FEDF-9DAD-51B7-9D8B-3A8797E51088

##### Material examined.

Argentina • 3 male macropters, 1 female macropter, Chaco, El Paranacito, 21-XII-2021, on *Pontederiarotundifolia*, Sosa-Salinas cols. (MLP); Bolivia • 1 male macropter, labeled 11.862, col. Berg (MLP).

##### Distribution.

Argentina: Formosa, Chaco, and Corrientes provinces (Sosa et al. 2007b). BRAZIL: Pará and Mato Grosso do Sul States (Muir, 1926). New record: Bolivia.

##### Biological aspects.

Adults and nymphs of *M.iphigeniae* were found abundantly on *P.azurea* and *P.crassipes* in northeastern Argentina (La Plata Basin, subregion Iberá System) and on *P.parviflora* Alexander in Brazil (Pantanal, subregion Paraguay River). *Pontederiarotundifolia* L.f. is a new record of host plant. Specimens were found abundantly on a small mat of *P.rotundifolia* plants stranded on the shore of a stream.

### ﻿Key to *Megamelus* species from South America (modified from Mariani et al. 2013)

*M.davisi has been included in this key because of its morphological similarity to *M.serpentinus* (Fig. 7). All the South American species here included are illustrated in Fig. 10.

**Table:** 

1	Male anal segment with 2 processes arising ventrally near anterior margin, caudally directed	**2**
–	Male anal segment with 2 processes arising ventrally near posterior margin or without processes	**4**
2(1)	Very short anal processes, 1/5 length of segment. Aedeagus globular at base, with long, basad-directed apical process closely curved towards the left. Ovipositor short and curved, not overpassing anal segment. Mostly brown, paler on frons disc, fore wing hyaline with infuscated marks on clavus, axillar and apical cells. Submedian frontal carina forked far from fastigium. Spur broadly rounded, with 18 fine teeth (Sosa et al. 2007b: figs 45–58)	***M.timehri* Muir**
–	Long anal processes, as long as or longer than the segment. Aedeagus not globular at base. Ovipositor long, overpassing anal segment	**3**
3(2)	Aedeagus flat with narrow twisted apex (Beamer 1955: 32, pl. 1, fig. 1). Dark brown, with the lobes of pygofer black; carinae, veins and lined marks of abdomen usually paler; forewings whitish, pale veins with darkish pigmentation on costal and commissural area of corium; tegmina castaneous with pale veins. Submedian frontal carina forked at the apex of fastigium. Spur large, foliaceous, oblong	M.davisi* Van Duzee
–	Aedeagus tubular, short, strongly narrow at base, with thin, short, straight process curved at apex (Fig. 5C, D). Dark brown, with distinctive yellowish to white marks bordering most of the sclerites of the body; fore wing amber, pale brown veins, with distinctive fuscous marks on central nodal and apical veins on membrane and clavus; tegmina amber, with fuscous transversal marks from axillar angle to clavus. Submedian frontal carina forked on vertex far from fastigium (Fig. 4A). Spur long and broad, apically truncated, with 17 or 18 large lateral teeth (Fig. 4G)	***M.serpentinus* sp. nov.**
4(1)	Anal segment with curved or folded processes near the posterior margin	**5**
–	Anal segment without processes	**7**
5(4)	Anal processes long, folded in 1/2 and parallel-sided; inner lobes of pygofer without process between them; aedeagus very long, slender, tubular; with long, thin, spine-like process closely curved to left. Ovipositor short, reaching anal segment at base. Brown, frons paler with small irregular darker spots on base, and narrow, irregular, blackish and whitish stripes toward apex; tegmina pale brown with dark spots on claval margin. Spur small, with 10–13 teeth (Mariani et al. 2013: figs 1–14)	***M.nigrifasciatus* Mariani & Remes Lenicov**
–	Anal processes short and curved, apically convergent or oppositely directed; inner lobes of pygofer with a process between them. Ovipositor straight and long	**6**
6(5)	Anal processes symmetrical, convergent apically; pygofer with sclerotized area bearing pair of small, sharply pointed processes between inner lobes. Brown, frons with narrow pale stripe on apex; fore wing hyaline with only 1 fuscous mark on apex of clavus. Spur long, with 15–20 teeth (Sosa et al. 2007b: figs 2–15)	***M.bellicus* Remes Lenicov & Sosa**
–	Anal processes asymmetrical, oppositely directed apically; pygofer with lobe-like process between inner lobes. Pale brown, frons uniformly colored; fore wing heavily infuscated on clavus and apical area. Spur large, wide, with 20–22 teeth (Sosa et al. 2007b: figs. 17–30)	***M.electrae* Muir**
7(4)	Pygofer inner lobes truncated; aedeagus long, with 2 apical lateral processes. Ovipositor long, overpassing anal segment at base, with conspicuous truncated and dorsally denticulated teeth. Brown, frons with yellowish spots on fastigium and 2 paler transverse stripes. Spur long, with 13 or 14 small and big teeth alternated on the basal 1/2 and larger toward the apex (Sosa et al. 2004: figs 1–14)	***M.scutellaris* Berg**
–	Pygofer inner lobes rounded, sinuous or subtriangular in outline; aedeagus with a single apical process, simple or bifurcated. Ovipositor short, reaching anal segment at base	**8**
8(7)	Genital styles with apex wide and truncated (Fig. 2E), aedeagus with apical process forked at base (Fig. 2D), pygofer inner lobes subtriangular in outline (Fig. 2A). Body broad and flattened, with vertex broad, subquadrate, and apically rounded; submedian carina forking dorsally just at the fastigium, which is angulated in lateral view; frons subcircular. Dull dark brown mottled with paler spots on apex of vertex and frons disc, and stripe on epistomal area extending towards base of gena (Fig. 1B). Spur small with 8 or 9 black-tipped sharp teeth (Fig. 1D)	***M.delticus* sp. nov.**
–	Genital styles with hook-like apex, aedeagus with single apical process, pygofer inner lobes rounded or sinuous in outline	**9**
9(8)	Pygofer inner lobes rounded in outline; aedeagus with long spine-like subapical process curved downwards on the left; genital styles strongly flexed inwards midway. Brown, frons with 2 paler but wide transverse stripes; forewings hyaline with veins and apical cells infuscated, tegmina amber with dark spots on apical and claval margin. Long antennal basal segment. Spur short, with 20 small teeth (Mariani et al. 2013: figs. 15–28)	***M.maculipes* (Berg)**
–	Pygofer inner lobes sinuous in outline; aedeagus bearing 1 long process, expanded proximally and curved upwards apically on the right; genital styles straight, curved at apex. Pale brown, uniformly colored; forewings hyaline with fuscous marks on apex of clavus, along median and cross veins, and on 2 apical cells. Short antennal basal segment. Spur long, with 19–24 regular, large teeth (Sosa et al. 2007b: figs 31–44)	***M.iphigeniae* Muir**

### ﻿Phylogenetic analyses

**Figure 9.**
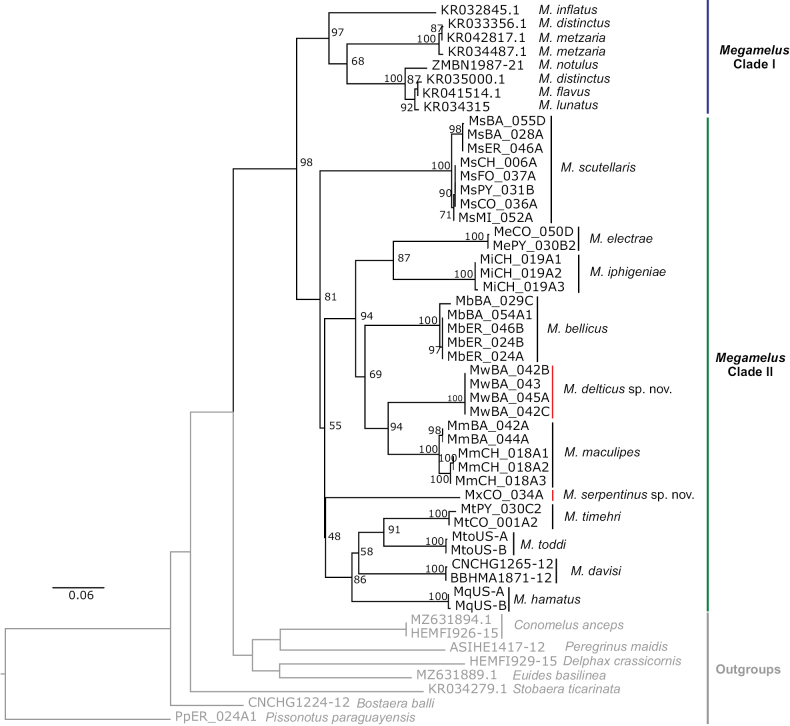
Maximum likelihood (ML) topology (likelihood, -6249.51) resulting from the analysis of the COI gene. UFBootstrap node support values are indicated above each node. Branch length represents the number of nucleotide substitutions per nucleotide site (scale bar = 0.06 substitutions per site). Newly described species are marked with a red line. Outgroups are shown in pale gray color.

The phylogenetic relationships among several species of the genus *Megamelus* were reconstructed by means of ML based on a 658 pb fragment of the mitochondrial COI gene (Fig. 9, Suppl. material 3). We retrieved two main clades: one comprising most Holarctic species (Clade I) and one containing the South American species and three North American species: *M.davisi*, *M.hamatus*, *and M.toddi* (Clade II). Within Clade II, all species are clearly delimited, with high bootstrap values for external nodes. However, these values decrease for some older diversification events. *Megamelusscutellaris* appears as a sister group of the remainder of Clade II, which is then separated into two subclades: one comprises *M.timehri*, *M.toddi*, *M.davisi*, *M.hamatus*, and *M.serpentinus*. The other one contains the remaining South American *Megamelus* species and is subdivided into two more clades, one composed of *M.electrae* and *M.iphigeniae*, and the other one including *M.maculipes*, *M.delticus*, and *M.bellicus*.

## ﻿Discussion

**Figure 10.**
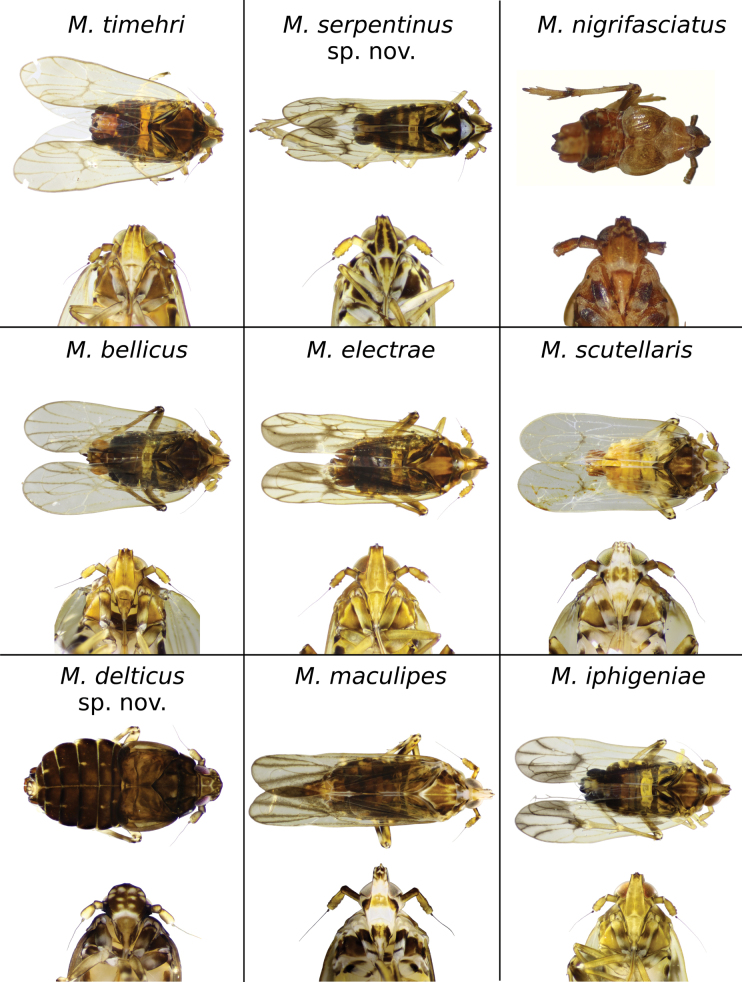
Dorsal and ventral view of the head of the nine South American *Megamelus* species. Macropterous males are shown for all species except for *M.delticus* sp. nov. and *M.nigrifasciatus*, for which only the brachypter is known.

In the present study we describe two new species, *Megamelusdelticus* sp. nov. and *Megamelusserpentinus* sp. nov., increasing to nine the number of South American *Megamelus* species known to date and to 33 worldwide (Fig. 10). The identity of these species, previously defined through the analysis of morphological characters, is now supported by genetic data, which also allowed us to investigate the phylogenetic relationships among the 18 species of the genus. Lastly, this revision of the South American species enabled us to reassess the traits considered typical of the genus and to expand the existing key.

The genus *Megamelus* was originally described mainly based on the coloration pattern, head morphology (narrow vertex and trapezoidal frons), carinae of pro- and mesothorax, and the relative length of the metatarsomeres (Fieber 1866). In addition, the lobed appearance of the pygofer and its genito-anal structures were considered by Muir (1926) and later emphasized by Beamer (1955) as the main diagnostic features for this genus. On a first approach, the case of *M.delticus* sp. nov. seems to present a contradiction. Although some attributes of this species, such as a greatly flattened and oval body, a broad and short head, angled fastigium when viewed laterally, and small eyes, are inconsistent with the ones of *Megamelus*, its pygofer and genito-anal structures do match with those of the genus. Furthermore, its classification within the genus was confirmed by our phylogenetic analysis. These combined results support the notion that the lobed appearance of the pygofer is the distinctive morphological feature for characterizing the genus, while other traits might exhibit more variation than previously thought.

Previous studies have explored the phylogenetic relationships among the Delphacidae using morphological characters (Asche 1985), genetic markers (Dijkstra et al. 2003; Huang et al. 2017; Bucher et al. 2023) or a combination of both (Urban et al. 2010). Some of these studies included the genus *Megamelus*, and placed it as a sister group of the genus *Conomelus*. However, phylogenetic relationships within the genus have not yet been explored. Here, newly generated COI data along with sequences retrieved from public databases were used to reconstruct the relationship among the *Megamelus* sp. for the first time. These analyses confirm that the genus constitutes a well-supported clade and its monophyly is corroborated by the inclusion of several sequences of closely related delphacid genera. Moreover, the identity of the South American *Megamelus* species, including the two described in this article, is supported by this analysis. However, this is not the case for some sequences belonging to North American species, whose placement in the tree could be the result of a misidentification of the specimens deposited in databases and should be corroborated. Although the analysis of the COI gene sequences yielded a topology with little resolution at some diversification events, it allowed us to shed some light on the relationships among the species of the genus. Further investigation including more species and the use of more powerful genetic markers for solving deep nodes are required in order to fully clarify the relationships among the *Megamelus*.

While many delphacid genera are monophagous or oligophagous, typically feeding on hosts within the same genus or family (e.g., *Prokelisia* (Van Duzee, 1897) on *Spartina* spp. (Bartlett 2020), *Kelisia* Fieber, 1878 on Cyperaceae (Bartlett and Wheeler 2007)), the genus *Megamelus* has a much broader range of hosts, including both monocots and dicots. However, each *Megamelus* species appears to be monophagous or oligophagous, with the common trait that their hosts are found in wetland habitats. South American *Megamelus* are mostly associated with macrophytes: *M.scutellaris*, *M.electrae*, *M.bellicus*, *M.iphigeniae*, and *M.serpentinus* sp. nov. feeding on plants of the genus *Pontederia* (Pontederiaceae); *M.maculipes* on *Echinodorus* sp. (Alismataceae), and *M.timehri* on *Nymphoidesindica* (Menyanthaceae). The exceptions to this seem to be *M.nigrifasciatus* (Mariani et al. 2013) and *M.delticus* sp. nov., both of which have been recorded on *Eryngium* sp. (Apiaceae), a plant that grows on the higher areas of river banks that can remain protected from floods for long periods of time. However, plants in this genus tend to accumulate rain water in the center of the mat, where both species have been found, which suggests that a close proximity to a water source is a common factor for all known species.

Host plant use seems to be one of the main forces promoting interspecific divergence in herbivorous insects (Dijkstra et al. 2003; Poveda-Martínez et al. 2020, 2022) and this should be interpreted by integrating different approaches. From a morphological standpoint, host plants have been proposed to exert a strong selective pressure in certain structures in Delphacidae, such as the ovipositor (Wallner and Bartlett 2019) and the spur (Markevich et al. 2021). For example, in this study we observed that South American *Megamelus* species with a long ovipositor and gonapophysis IX serrated in > 1/2 of its dorsal and ventral-apical margin, and with a wide foliaceous spur with numerous teeth, have the Pontederiaceae as host plants. Meanwhile, those species that live and feed on *Eryngium* sp., *M.nigrifasciatus*, and *M.delticus* sp. nov., have short gonapophysis with rounded denticulation and a spur considerably reduced in terms of size and teeth number (≤ 13), showing a notable adaptation to the microhabitat offered by their host. For *M.maculipes* and *M.timehri*, feeding on *Echinodorus* sp. and *Nymphoidesindica*, respectively, the association between host plant and these morphological traits is not clear. Moreover, we also noticed that *M.timehri*, *M.toddi*, and *M.davisi*, which comprise a clade of species in the phylogenetic tree, feed on rooted aquatic plants with floating leaves, living in close proximity with the water surface. Despite the low nodal support of this clade, we hypothesize that these species might have derived from an ancestor which fed on a macrophyte with a similar life form. In fact, these species possess a large, foliaceous spur which might allow them to walk on the water surface (Van Duzee 1897; Sosa et al. 2007b; Suppl. material 4 for *M.toddi*).

The relatively recent interest in the genus *Megamelus* due to the use of *M.scutellaris* as a biological control agent (Tipping et al. 2014; Coetzee et al. 2022) motivated field surveys and studies that contributed to the knowledge on this group of planthoppers and on its diversity in the Neotropics. Further studies including multidisciplinary approaches that combine morphological, ecological, and genetic information, will allow a better understanding of the evolutionary history of these species and their relationships with their host plants and their environment.

## Supplementary Material

XML Treatment for Megamelus delticus

XML Treatment for Megamelus serpentinus

XML Treatment for Megamelus timehri

XML Treatment for Megamelus iphigeniae

## ﻿Additional information

### Author ORCIDs

Nicolas A. Salinas https://orcid.org/0000-0002-1055-4475

Roxana Mariani https://orcid.org/0000-0002-0066-6692

Ana M. Marino de Remes Lenicov https://orcid.org/0000-0001-8678-5408

Marcela S. Rodriguero https://orcid.org/0000-0001-5967-2045

Alejandro J. Sosa https://orcid.org/0000-0002-1680-8712

### Data availability

All of the data that support the findings of this study are available in the main text or Supplementary Information.

## Supplementary materials

Supplementary material 1 Collection data and accession number of genetic samples Data type xlsx Explanation note Collection data and accession number of genetic samples, including geographical coordinates, collection date, host plant and GenBank accession numbers. https://binary.pensoft.net/file/1228500This dataset is made available under the Open Database License (http://opendatacommons.org/licenses/odbl/1.0/). The Open Database License (ODbL) is a license agreement intended to allow users to freely share, modify, and use this Dataset while maintaining this same freedom for others, provided that the original source and author(s) are credited.Author: Nicolas A. Salinas

Supplementary material 2 Collection data of two new species of *Megamelus* from South America Data type xlsx Explanation note Collection data of two new species of *Megamelus* from South America, including geographical coordinates, host plants, and collection date. https://binary.pensoft.net/file/1228501This dataset is made available under the Open Database License (http://opendatacommons.org/licenses/odbl/1.0/). The Open Database License (ODbL) is a license agreement intended to allow users to freely share, modify, and use this Dataset while maintaining this same freedom for others, provided that the original source and author(s) are credited.Author: Nicolas A. Salinas

Supplementary material 3 Phylogenetic tree in Newick format Data type tree Explanation note Phylogenetic tree of the genus *Megamelus* in Newick format. https://binary.pensoft.net/file/1228502This dataset is made available under the Open Database License (http://opendatacommons.org/licenses/odbl/1.0/). The Open Database License (ODbL) is a license agreement intended to allow users to freely share, modify, and use this Dataset while maintaining this same freedom for others, provided that the original source and author(s) are credited.Author: Nicolas A. Salinas

Supplementary material 4 Detail of the hind leg of *M.toddi* (specimen sample code MtoUS-A), showing the foliaceous shape of the calcar Data type docx Explanation note Hind leg with detailed of spur of *Megamelustoddi*. https://binary.pensoft.net/file/1228503This dataset is made available under the Open Database License (http://opendatacommons.org/licenses/odbl/1.0/). The Open Database License (ODbL) is a license agreement intended to allow users to freely share, modify, and use this Dataset while maintaining this same freedom for others, provided that the original source and author(s) are credited.Nicolas A. Salinas, Roxana Mariani, Ana M. Marino de Remes Lenicov, Alejandro J. Sosa
