# Supplementary material for: ﻿Phylogeny of the planthopper genus Megamelus (Hemiptera, Delphacidae), with the description of two new species from South America
Source: Zookeys. 2025 Jan 21;1224:29–54. doi: 10.3897/zookeys.1224.135596 (PMC11775577; doi:10.3897/zookeys.1224.135596)
Supplement: Supplementary material 4 — Detail of the hind leg of M.toddi (specimen sample code MtoUS-A), showing the foliaceous shape of the calcar [file zookeys-1224-029_article-135596__-s004.docx]

**Supplementary material 4**


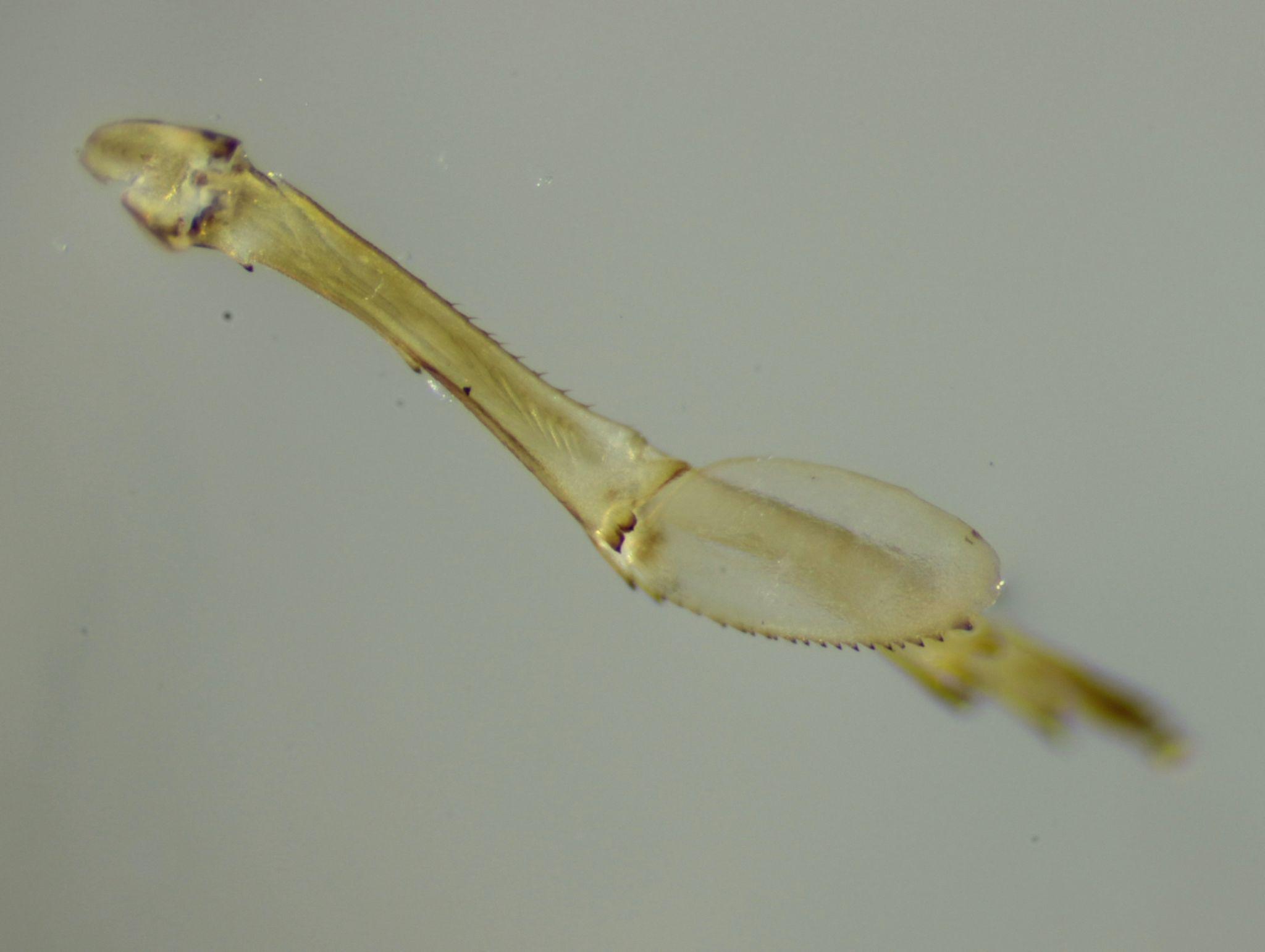


Detail of the hind leg of *M. toddi* (specimen sample code MtoUS-A), showing the foliaceous shape of the calcar.
